# Multiomics analyses of human colorectal cancer reveal changes in mitochondrial metabolism associated with chemotherapy resistance

**DOI:** 10.3389/fonc.2025.1625797

**Published:** 2025-11-10

**Authors:** Shiyi Chen, Qian Li, Wei Zheng

**Affiliations:** 1 Department of Pathology, Zhongshan Hospital of Xiamen University, School of Medicine, Xiamen University, Xiamen, China; 2 School of Medicine, Xiamen University, Xiamen, China

**Keywords:** proteomics, metabolomics, colorectal cancer, chemotherapy resistance, mitochondrial metabolism

## Abstract

**Background:**

Mitochondria are essential organelles involved in energy production, cellular metabolism, and signal transduction. They have important impacts on tumorigenesis and cancer progression. Nevertheless, the associations between mitochondrial metabolic processes and chemotherapy resistance in colorectal cancer (CRC) are not well understood.

**Methods:**

We generated a chemotherapy-resistant colorectal cancer cell line, HCT-15/DOX, via doxorubicin (DOX) induction. We then performed proteomic and metabolomic analyses via LC-MS/MS technology on both the parental and the DOX-resistant cell lines. Additionally, transmission electron microscopy was used to examine changes in mitochondrial morphology between the two cell lines.

**Results:**

The results revealed significant dysregulation of 185 proteins and 1099 metabolites in HCT-15/DOX cells relative to parental cells, highlighting the impact of chemotherapy resistance on cellular processes. The key functional proteins that were identified included upregulated SDHA, BCKDHB, CRYZ, NUDT6, CPT1A, and POLG, and downregulated CRAT, FDPS, SFXN1, and ATAD3B. Additionally, through combined multiomics pathway enrichment analysis, pyrimidine metabolism, purine metabolism, ascorbate and aldarate metabolism, propanoate metabolism, and the citrate cycle (TCA cycle) were identified as important metabolic processes associated with CRC chemotherapy resistance. Transmission electron microscopy analysis revealed that HCT-15/DOX cells had increased mitochondrial number, length, and area.

**Conclusions:**

This research highlights notable differences in mitochondrial morphology and diverse mitochondrial metabolic functions between parental and DOX-resistant HCT-15 CRC cells. The findings of the present study provide insights into the mitochondrial metabolic changes associated with CRC chemotherapy resistance, offering valuable insights into the mechanisms underlying these changes and identifying potential therapeutic targets for addressing CRC chemotherapy resistance.

## 1 Introduction

Colorectal cancer (CRC) is considered the third most commonly diagnosed cancer and the second primary cause of cancer-related death globally (1). It is projected that by 2040, the burden of CRC will add 3 million new cases and 1.6 million deaths annually (2). The current treatment options for CRC include endoscopic and surgical resection, preoperative radiotherapy, systemic therapy, local ablation therapy, palliative chemotherapy, targeted therapy, and immunotherapy (3). Chemotherapy is utilized for preoperative downstaging and serves as a critical systemic treatment for metastatic CRC. However, the resistance of CRC cells to chemotherapy can result in treatment failure (4). Research indicates that the mechanisms of chemotherapy resistance include factors such as increased drug efflux, tumor heterogeneity, increased DNA repair capacity, the tumor microenvironment, and genetic factors, including gene mutations, amplifications, and epigenetic changes (5, 6).

Mitochondria are vital organelles that are engaged in energy production, cellular metabolism, and signal transduction, and they have a vital impact on tumorigenesis and cancer progression (7). Increased mitochondrial oxidative phosphorylation (OXPHOS) and ROS production can increase resistance to chemotherapy in breast cancer (8). The mitochondrial enzyme glucosylceramide synthase (GCS) plays a significant role in sphingolipid metabolism. In addition, it is highly expressed in drug-resistant cancer cells, contributing to chemotherapy resistance (9). Moreover, multiple studies have implicated glycolysis-related enzymes in mitochondria in the progression of chemotherapy resistance in tumors (10–14). Furthermore, the metabolic reprogramming from oxidative phosphorylation to lactate fermentation in drug-resistant tumor cells represents a prevalent phenotypic mechanism in cancer. This adaptive metabolic shift may constitute a common hallmark in cancer biology that could be exploited for therapeutic purposes to overcome chemoresistance (15). Nonetheless, the specific mechanisms linking mitochondrial metabolic function and chemotherapy resistance in CRC are not yet fully understood.

Doxorubicin (DOX) is common treatment modality for several types of cancer, including CRC. It is known to induce chemotherapy resistance in CRC cell lines (16–19). This study developed a DOX-resistant colorectal cancer cell line called HCT-15/DOX. We conducted proteomic and metabolomic analyses via LC-MS/MS technology on both the original HCT-15 cell line and the DOX-resistant HCT-15/DOX cell line. We applied transmission electron microscopy to study changes in mitochondrial morphology. We analyzed the omics data and mitochondrial morphological parameters of the two groups of cells and found that, compared with the parental cells, the chemoresistant CRC cell lines presented different mitochondrial morphologies and metabolic patterns. This discovery will improve our understanding of the mechanisms underlying mitochondrial metabolic changes linked to CRC chemotherapy resistance and will help identify potential therapeutic targets for treating CRC chemotherapy resistance.

## 2 Materials and methods

### 2.1 Compounds

Oligomycin (TargetMol, USA), an antifungal antibiotic, is an inhibitor of H+-ATP synthase. Oligomycin can block oxidative phosphorylation and the electron transport chain. The compounds were dissolved in dimethyl sulfoxide (DMSO; MP Biomedicals, Solon, OH, USA). In addition, the concentration of DMSO in the cell cultures was ensured to be less than 0.1% during the experiments.

### 2.2 Cell culture and establishment of a doxorubicin-resistant cell line

The human colorectal cancer cell line HCT-15 was acquired from the Cell Bank of the Chinese Academy of Sciences. To establish a doxorubicin-resistant subline, a high-dose pulse induction method was employed. HCT-15 cells in the logarithmic growth phase were seeded into culture dishes. Once the cells reached 70%–80% confluence, doxorubicin (MedChemExpress, Monmouth Junction, NJ, USA) was supplemented at a final concentration of 5 μg/mL. After 2 h of drug exposure, the medium containing the drug was discarded, and viable cells were collected and seeded into new culture dishes. Once the cells recovered and grew to 70–80% confluence, the procedure was repeated. This process was continued until the cell death rate under a doxorubicin concentration of 0.4–0.5 μg/mL was less than 5%. The resulting resistant cell line was designated HCT-15/DOX. Both HCT-15 and HCT-15/DOX cells were cultivated in RPMI 1640 medium (VivaCell, Shanghai, China) supplemented with 10% fetal bovine serum (VivaCell, Shanghai, China) and 1% penicillin/streptomycin (VivaCell, Shanghai, China).

### 2.3 Cell counting kit-8 assay

Parental and doxorubicin-resistant HCT-15 cells were seeded into 96-well plates at a density of 5 × 10^3^ cells per well and cultivated for 12 h. To identify the half-maximal inhibitory concentration (IC50) of doxorubicin, each cell line was subjected to treatment with varying concentrations of doxorubicin for 24 h. When the cells were washed three times with PBS, 100 μL of fresh medium and 10 μL of Cell Counting Kit-8 (CCK-8) reagent (TargetMol, USA) were added to each well. The cells were subsequently incubated at 37 °C for an additional 2 h. A microplate reader was used to measure the absorbance at 450 nm.

### 2.4 Transmission electron microscopy observation of mitochondria

The cells were collected via centrifugation. The cell pellet was resuspended in electron microscope fixation solution, ensuring uniform mixing, and fixed at 4°C for 2–4 h. Following centrifugation, the supernatant was discarded, and 0.1 M phosphate buffer (PB, pH 7.4) was added. The mixture was washed for 3 min, followed by centrifugation. This washing step was repeated three times. A 1% agarose solution was preheated, dissolved, and then slightly cooled before being added to an EP tube. Prior to agarose solidification, the pellet was removed with tweezers and embedded in agarose. The samples were fixed in 1% osmium tetroxide (OsO_4_) prepared with 0.1 M phosphate buffer (PB, pH 7.4) at room temperature in the dark for 2 h. In addition, dehydration was carried out with a graded ethanol series, with each step lasting 20 min. The samples were then infiltrated and embedded in resin, and ultrathin sections (60–80 nm) were cut via an ultramicrotome. Later, the sections were gathered on 150-mesh square copper grids with Formvar support film. The grids were stained with 2% uranyl acetate saturated in ethanol in the dark and then air-dried at room temperature overnight. Finally, a transmission electron microscope was used to observe the mitochondria. Moreover, images were gathered for analysis. Mitochondrial measurements were conducted with ImageJ software (version 1.54d).

### 2.5 Proteomics

#### 2.5.1 Protein extraction and digestion

In this study, SDT (4% SDS, 100 mM Tris-HCl, and 1 mM DTT, pH 7.6) buffer was used for sample lysis and protein extraction. To quantify the protein concentration, a BCA protein assay kit (Bio-Rad, USA) was used. Protein digestion by trypsin was carried out following the filter-aided sample preparation (FASP) procedure described by Matthias Mann. In addition, the digested peptides of each sample were desalted on C18 cartridges (Empore™ SPE Cartridges C18 (standard density), bed I.D. 7 mm, volume 3 mL, Sigma), concentrated by vacuum centrifugation, and then reconstituted in 40 µL of 0.1% (v/v) formic acid for peptide quantification (OD280).

#### 2.5.2 LC-MS/MS analysis

LC-MS/MS analysis was carried out on a timsTOF Pro mass spectrometer (Bruker) coupled to a NanoElute (Bruker Daltonics) for 60/120/240 min. Next, the peptides were loaded onto a reversed-phase trap column (Thermo Scientific Acclaim PepMap100, 100 μm*2 cm, nanoViper C18), which was associated with a C18-reversed-phase analytical column (Thermo Scientific Easy Column, 10 cm long, 75 μm inner diameter, 3 μm resin) in buffer A (0.1% formic acid) and separated with a linear gradient of buffer B (84% acetonitrile and 0.1% formic acid) at a flow rate of 300 nL/min regulated by IntelliFlow technology. Afterwards, the mass spectrometer was used in positive ion mode, and it gathered ion mobility MS spectra over a mass range of m/z 100–1700 and 1/k0 of 0.6–1.6 and subsequently carried out 10 cycles of PASEF MS/MS with a target intensity of 1.5k and a threshold of 2500. Moreover, active exclusion was enabled with a release time of 0.4 min.

#### 2.5.3 Identification and quantitation of proteins

For identification and quantitative analysis, MaxQuant 1.5.3.17 software was used to integrate and search the raw MS data for each sample.

### 2.6 Metabolomic

#### 2.6.1 Sample collection and preparation

A pipette was used to remove the culture medium from the cultured cells (~10^7^ cells per sample). The cells were subsequently rinsed with PBS at 37°C, after which the PBS was removed. A total of 800 μL of cold methanol/acetonitrile (1:1, v/v) was used to remove the protein and extract the metabolites. The mixture was collected into a new centrifuge tube and then subjected to centrifugation at 14000 ×g for 5 min with the aim of collecting the supernatant. A vacuum centrifuge was used to dry the supernatant. For LC-MS analysis, the samples were redissolved in 100 μL of acetonitrile/water (1:1, v/v) as the solvent. To monitor the stability and repeatability of instrument analysis, quality control (QC) samples were prepared by pooling 10 μL of each sample and combining it with the other samples. In addition, the QC samples were regularly inserted and then explored every five samples.

#### 2.6.2 LC-MS/MS analysis

This analysis was conducted with a UHPLC (1290 Infinity LC, Agilent Technologies) coupled to a quadrupole time-of-flight (AB Sciex TripleTOF 6600) at Shanghai Applied Protein Technology Co., Ltd.

For HILIC separation, samples were explored with a 2.1 mm × 100 mm ACQUITY UPLC BEH 1.7 µm column (Waters, Ireland). In both the positive and negative ESI modes, the mobile phase included A = 25 mM ammonium acetate and 25 mM ammonium hydroxide in water and B = acetonitrile. The gradient was 85% B for 1 min and was linearly decreased to 65% in 11 min. Subsequently, it was lowered to 40% in 0.1 min and maintained for 4 min, followed by an increase to 85% in 0.1 min, with a 5 min re-equilibration period being used. The ESI source conditions were as follows: ion source gas 1 (Gas1) of 60, ion source gas 2 (Gas2) of 60, curtain gas (CUR) of 30, source temperature of 600°C, and ion spray voltage floating (ISVF) of ±5500 V. The instrument was adopted for obtaining masses in the m/z range of 60–1000 Da in MS-only acquisition. Moreover, the accumulation time for the TOF MS scan was defined as 0.20 s/spectra. For auto MS/MS acquisition, the instrument was set to obtain masses in the m/z range of 25–1000 Da. Moreover, the accumulation time for the product ion scan was defined as 0.05 s/spectra. The product ion scan is obtained with information-dependent acquisition (IDA) in high-sensitivity mode. The parameters were as follows: the collision energy (CE) was fixed at 35 V with ±15 eV; the declustering potential (DP) was 60 V (+) and –60 V (–); and the isotopes within 4 Da were excluded, with the number of candidate ions to monitor per cycle being 10.

#### 2.6.3 Data processing

Before being imported into freely available XCMS software, the raw MS data were converted to MzXML files via ProteoWizard MSConvert. For peak picking, the following parameters were applied: centWave m/z = 10 ppm, peak width = c (10, 60), and prefilter = c (10, 100). In terms of peak grouping, bw = 5, mzwid = 0.025, and minfrac = 0.5 were applied. CAMERA (Collection of Algorithms of MEtabolite pRofile Annotation) was adopted for the annotation of isotopes and adducts. For the extracted ion features, only the variables that had over 50% of the nonzero measurement values in at least one group were maintained. The metabolites were identified through comparisons of the accuracy of the m/z values (<10 ppm) and the MS/MS spectra with an in-house database established with available authentic standards. With the KNN (K-nearest neighbor) method, the missing data were filled, and the extreme values were deleted. Finally, to ensure the parallelism between samples and metabolites, the total peak area of the data was normalized.

### 2.7 Data analysis for proteomics and metabolomics

Principal component analysis (PCA), Gene Ontology (GO) term enrichment analysis, Kyoto Encyclopedia of Genes and Genomes (KEGG) pathway enrichment analysis, volcano plots, Venn diagrams, and O2PLS analysis were carried out via OmicShare tools, which is a free online platform for performing data analysis (https://www.omicshare.com/tools). Metaboanalyst was used to perform joint pathway analysis. KM plotter (https://kmplot.com/analysis/) was employed to perform survival analysis.

### 2.8 Sulforhodamine B assay

The logarithmic-phase cells were collected, the cell suspension concentration was adjusted, and the cells were aliquoted into a 96-well plate at 100 μL per well. The plate was subjected to incubation at 37°C with 5% CO_2_ to allow for cell attachment and cultured for 6–24 h. Following incubation, the plate was removed, the culture medium was discarded, and the cells were rinsed 1–2 times with PBS. Subsequently, 100 μL of precooled fixative (Yeasen, Shanghai, China) was added to each well, and the mixture was incubated at room temperature for 5 min, followed by incubation at 4°C for 1 h. The supernatant was discarded, the mixture was rinsed three times with Wash Buffer 1 (Yeasen, Shanghai, China), and the mixture was air dried at room temperature. Then, 100 μL of staining solution (Yeasen, Shanghai, China) was added to each well, and the samples were incubated in the dark for 20 min (the plate was wrapped in aluminum foil and gently shaken on a horizontal or rocking shaker). After incubation, the staining solution was discarded, and the samples were rinsed five times with Wash Buffer 2 (Yeasen, Shanghai, China) (to prevent leakage of the staining solution from the cells), ensuring that the residual staining solution was thoroughly removed and air-dried at room temperature. Solubilization buffer (Yeasen, Shanghai, China) (200 µL) was added to each well, and the mixture was incubated in the dark for 30 min (the plate was wrapped in aluminum foil and gently shaken on a horizontal or rock-ing shaker). Finally, the absorbance was measured at a wavelength of 515 nm.

### 2.9 Mitochondrial DNA copies

The number of mtDNA copies was determined by targeting four distinct genes, namely, NADH dehydrogenase subunit 1 (ND1), solute carrier organic anion transporter family member 2B1 (SLCO2B1), NADH dehydrogenase subunit 5 (ND5), and serpin family A member 1 (SERPINA1), using the Human mtDNA Monitoring Primer Set (Cat. #7246; Takara Bio, Tokyo, Japan). Genomic DNA was extracted from the cell samples using a DNA extraction kit (Cat. #RK30110; ABclonal, Wuhan, China). For the subsequent reaction, a mixture was prepared containing the extracted genomic DNA, four specific primers from the Human mtDNA Monitoring Primer Set, and a PCR enzyme (SYBR Premix Ex Taq™ II, Cat. #RR820A; Takara Bio, Tokyo, Japan). In addition, quantitative PCR amplification was conducted with a Roche LightCycler^®^ 480 system under the following thermal cycling conditions: an initial denaturation step at 95°C for 30 s, followed by 40 cycles of denaturation at 95°C for 5 s and annealing/extension at 60°C for 30 s. Then, the mtDNA copy number was calculated as the mean of 2ΔCt values derived from the cycle threshold (Ct) differences between ND1/SLCO2B1 and ND5/SERPINA1.

## 3 Results

### 3.1 Establishment of the doxorubicin-resistant colorectal cancer cell line HCT-15/DOX

**Figure 1:**
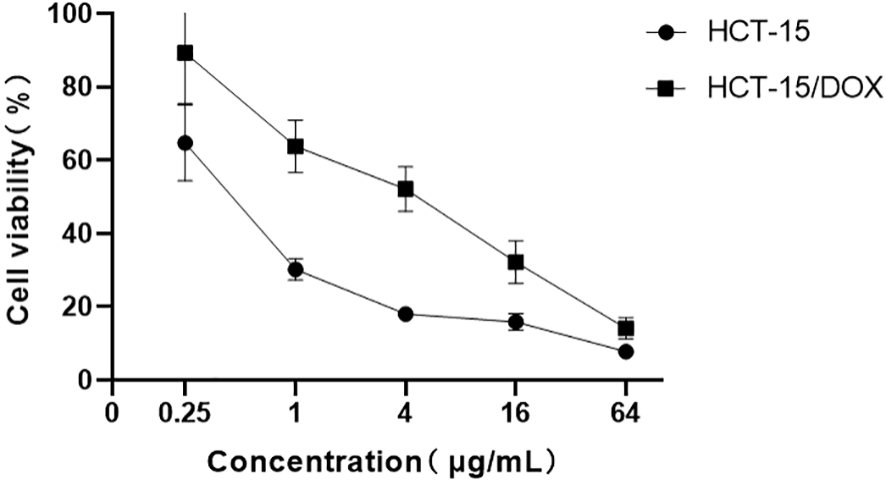
The viability of HCT-15 and HCT-15/DOX cells subjected to treatment with doxorubicin was assessed through the CCK-8 assay. Compared with parental cells, HCT-15/DOX cells were less sensitive to doxorubicin. Both HCT-15 and HCT-15/DOX cells were exposed to doxorubicin at concentrations of 0, 0.25, 1, 4, 16, and 64 µg/mL for 48 h. Afterward, the CCK8 assay was performed. Points indicate mean values; lines represent standard deviations (n = 5).

This study established a colorectal cancer cell line (HCT-15/DOX) that is resistant to doxorubicin (DOX) using a high-dose induction method. To assess the sensitivity of both parental and resistant HCT-15 cells to doxorubicin, we used a Cell Counting Kit-8 (CCK8) cell viability assay. The results revealed that the half-maximal inhibitory concentrations (IC50s) of DOX for HCT-15/DOX cells and parental cells were 4.27 ± 2.11 and 0.45 ± 0.18 µg/mL, respectively. Compared with the parental cells, the HCT-15/DOX cells exhibited a 9.49-fold increase in the IC50 value for doxorubicin (p < 0.01), indicating a markedly enhanced drug resistance. The data presented in **Figure 1** and **Table 1** clearly indicate that the HCT-15/DOX cell line, established as part of this study, has stable chemoresistance to doxorubicin.

**Table 1:** IC50 values of doxorubicin obtained from HCT-15 and HCT-15/DOX cells after 48 h of treatment. The values represent the means ± SDs of five independent experiments (n = 5). Student’s t test was used to calculate significance.

Cell line	IC50 of DOX (µg/mL)	Fold difference
HCT-15	0.45 ± 0.18	
HCT-15/DOX	4.27 ± 2.11	9.49**

**p < 0.01.

### 3.2 Increased mitochondrial number and altered mitochondrial morphology in HCT-15/DOX cells

**Figure 2:**
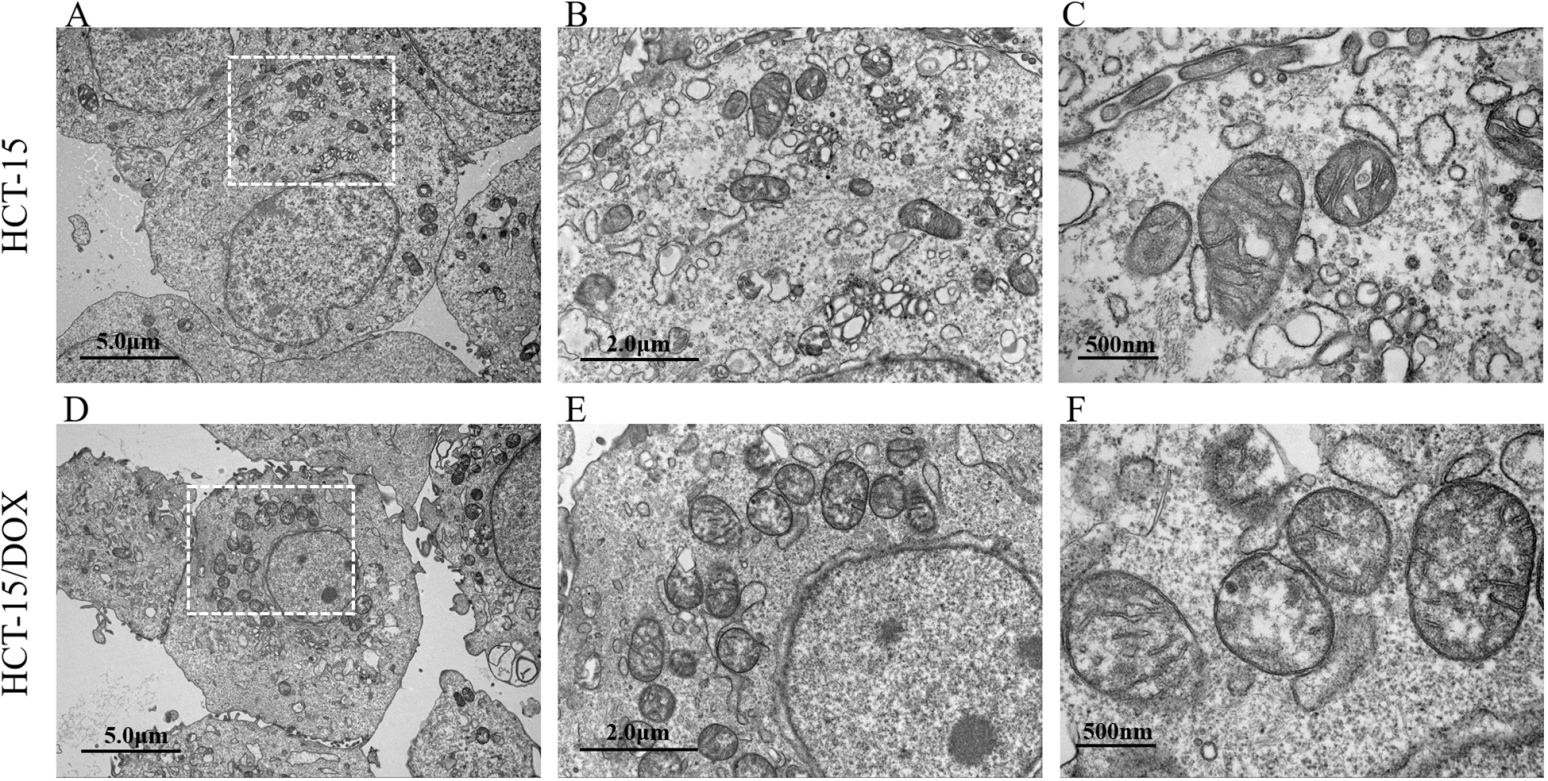
Representative electron microscopy images of mitochondria in HCT-15 **(A–C)** and HCT-15/DOX **(D–F)** cells. Scale bars represent 5 µm **(A, D)**, 2 µm **(B, E)**, and 500 nm **(C, F)**.

We conducted electron microscopy analysis on fixed samples of both HCT-15 and HCT-15/DOX cells. The transmission electron microscopy images revealed an increased number of mitochondria in HCT-15/DOX cells compared with parental HCT-15 cells (As shown in **Figures 2A, D**; **Supplementary Figures S1A-S1F**; **Table 2**). Additionally, electron microscope images at higher magnification were used to further analyze the morphological characteristics of the mitochondria (as shown in **Figures 2B, C, E, F**).

**Table 2:** Electron microscopic analysis of mitochondrial morphology in HCT-15 and HCT-15/DOX cells. To quantitatively assess mitochondrial characteristics, we randomly selected five cells each from the HCT-15 and HCT-15/DOX cell lines for mitochondrial counting. Additionally, we measured the maximum length and cross-sectional area of ten mitochondria per cell line for statistical analysis.

Statistics	Mitochondria number		Mitochondrial maximum length (μm)		Mitochondrial area (μm^2^)	
	HCT-15	HCT-15/DOX	HCT-15	HCT-15/DOX	HCT-15	HCT-15/DOX
Mean	22.4	33	0.6735	0.8903	0.2974	0.4923
Standard Error	4.0373	5.9161	0.2555	0.1669	0.1565	0.1629
Student’s t test (P value)	0.0107*		0.0471*		0.0185*	

*P<0.05.

To calculate statistical significance, Student’s t test was adopted, and the analysis revealed that the number, maximum length, and area of mitochondria notably increased in HCT-15/DOX cells compared with those in parental HCT-15 cells (As shown in **Table 2**, **Supplementary Figures S2A-S2C**). To measure the number of mitochondria, the number of mitochondria in three randomly selected low-power fields was counted. To calculate the maximum mitochondrial length, the maximum length of 10 randomly selected mitochondria was measured. The mitochondrial area was determined by measuring the area of 10 randomly selected mitochondria. The number, maximum length (μm), and area (μm^2^) of mitochondria were measured via NIH ImageJ software.

### 3.3 Proteins involved in mitochondrial metabolism changes were differentially expressed in HCT-15 and HCT-15/DOX cells

**Figure 3:**
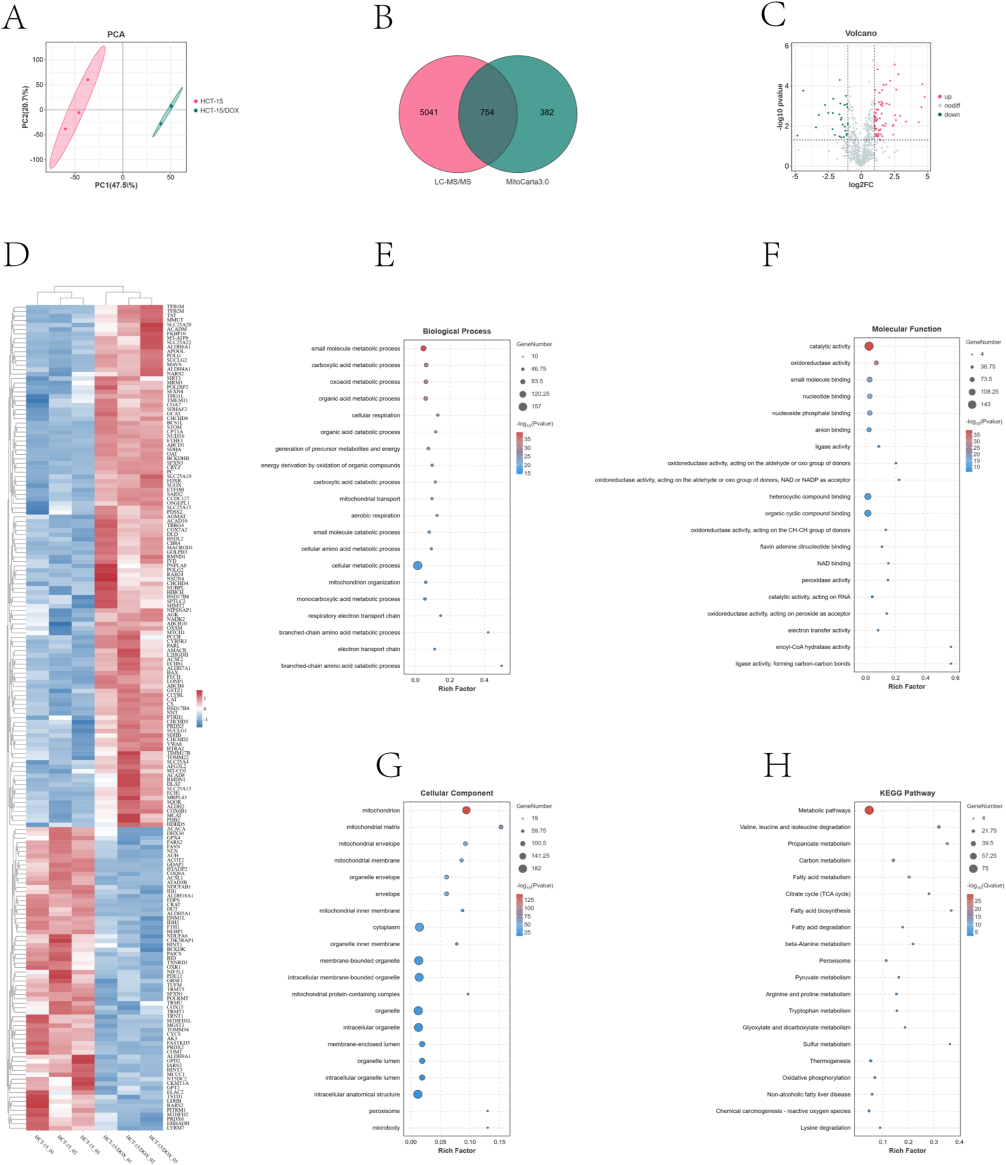
Differentially expressed proteins (DEPs) identified via proteomic analysis of HCT-15 and HCT-15/DOX cells. **(A)** PCA plot of the 5795 proteins detected in the proteomics analysis; **(B)** Venn diagram illustrating the intersection of 754 mitochondrial proteins identified through LC-MS/MS proteomics and the MitoCarta 3.0 database; **(C)** Volcano plot of the 754 mitochondrial proteins on the basis of Student’s t test, p values, and ratios of protein expression in HCT-15 and HCT-15/DOX cells. The green and red dots indicate the downregulated and upregulated proteins whose expression significantly differed, respectively. **(D)** Heatmap of the 185 differentially expressed mitochondrial proteins (DEPs). **(E-H)** GO enrichment analysis (BP\MF\CC) and KEGG pathway enrichment analysis of the 185 mitochondrial DEPs.

To investigate the mechanism by which CRC cells acquire drug resistance, we performed quantitative proteomics analysis of HCT-15/DOX and parental HCT-15 cells using LC-MS/MS. As shown in the analysis, 5795 proteins were detected (as shown in **Supplementary Table S3**). Principal component analysis (PCA) revealed significant differences between the two groups (**Figure 3A**). From the proteins identified in our proteomic analysis, we selected differentially expressed proteins (with a fold change ≥1.5 or ≤0.67 and a P value <0.05) for KEGG pathway analysis. The results revealed significant alterations in several pathways, including DNA replication, amino sugar and nucleotide sugar metabolism, glycolysis/gluconeogenesis, and fatty acid metabolism (**Supplementary Figure S3**).

To identify proteins specific to mitochondria, we referenced the human mammalian mitochondrial protein dataset from MitoCarta 3.0 (An Inventory of Mammalian Mitochondrial Proteins and Pathways) and identified 754 mitochondrial proteins (as shown in **Supplementary Table S4**, **Figures 3B, C**. Among them, 185 differentially expressed proteins (DEPs) were identified via specific screening criteria (fold change ≥ 1.5 or ≤ 0.67 and *p* < 0.05) as the cutoff threshold (**Supplementary Table S5**). This included 117 proteins whose expression was upregulated and 68 whose expression was downregulated. The key functional proteins that were identified included upregulated SDHA, BCKDHB, CRYZ, NUDT6, CPT1A, and POLG and downregulated CRAT, FDPS, SFXN1, and ATAD3B. A hierarchical clustering heatmap further revealed significant differences in the proteomic patterns between the chemotherapy-resistant and parental CRC cell lines (**Figure 3D**). We investigated the prognostic relevance of CPT1A and POLG expression in colorectal cancer (CRC) using publicly available oncology databases. Our analysis suggested that elevated expression levels of both CPT1A and POLG in CRC tissues were strongly associated with poor patient prognosis (**Supplementary Figures S4A, S4B**). POLG serves as the essential human mitochondrial DNA polymerase responsible for mitochondrial genome replication and repair. Given its significant upregulation in drug-resistant cells, we further validated the difference in mitochondrial DNA (mtDNA) copy number between resistant and parental cell lines, revealing a marked increase in mtDNA copy number in resistant cells compared with their parental counterparts (**Supplementary Figure S5**).

The functional characteristics of the DEPs were further explained through gene ontology (GO) enrichment analysis (**Figures 3E–G**). This analysis revealed three types: biological process (BP), molecular function (MF), and cellular component (CC). The biological processes of the DEPs were significantly enriched in small molecule metabolic processes, carboxylic acid metabolic processes, oxoacid metabolic processes, organic acid metabolic processes, cellular respiration, and organic acid catabolic processes. With respect to molecular function (MF), these proteins were shown to be involved primarily in catalytic activity, oxidoreductase activity, small molecule binding, nucleotide binding, and nucleoside phosphate binding. The proteins in the CC category were involved mainly in the mitochondrion, mitochondrial matrix, mitochondrial envelope, and mitochondrial membrane. According to the KEGG pathway analysis, these DEPs were obviously enriched in metabolic pathways: valine, leucine, and isoleucine degradation; propanoate metabolism; carbon metabolism; fatty acid metabolism; and the citrate cycle (TCA cycle) (**Figure 3H**).

### 3.4 Metabolomic profiles and functional alterations in HCT-15 and HCT-15/DOX cells

**Figure 4:**
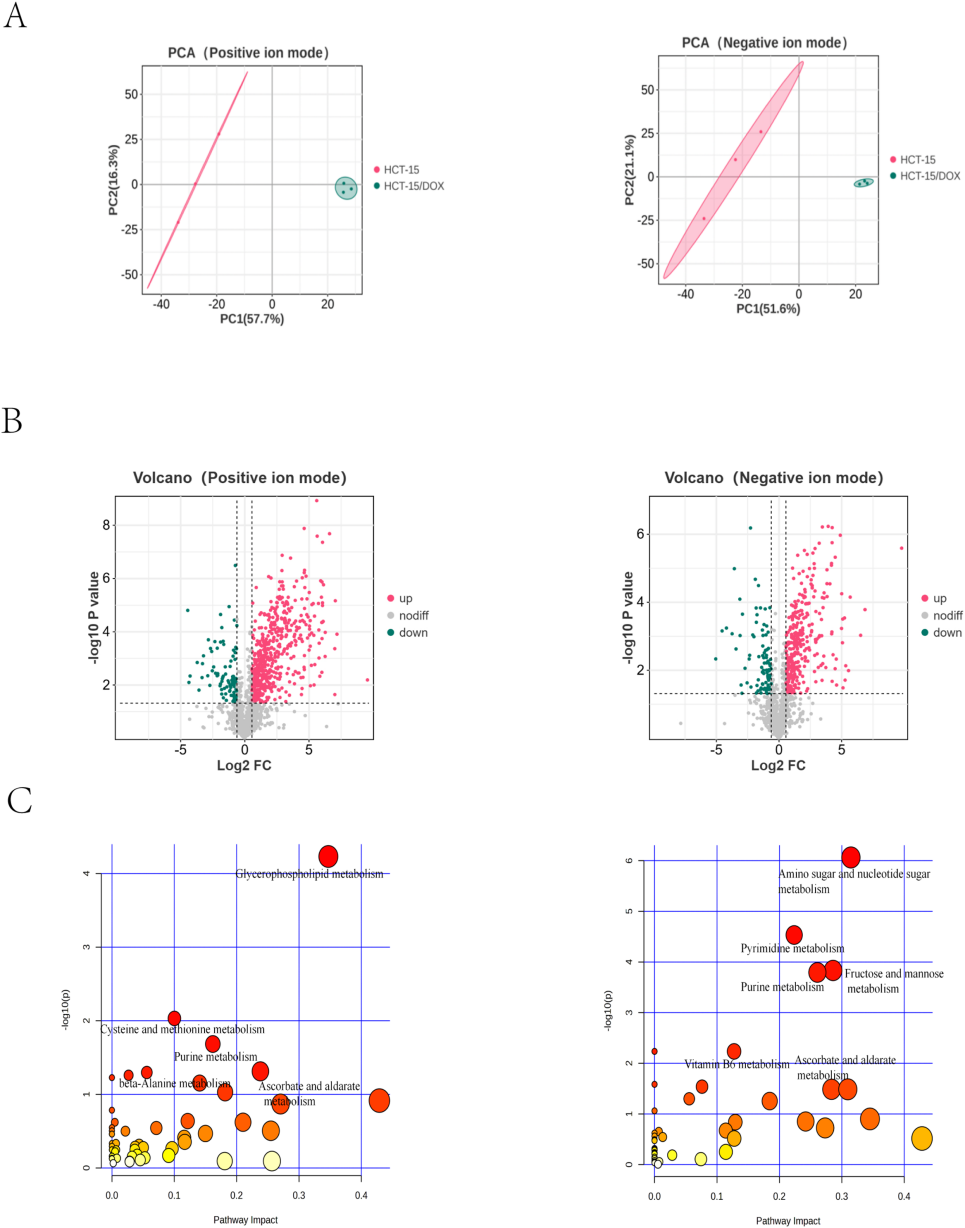
Differentially expressed metabolites identified via targeted metabolomics analysis of HCT-15 and HCT-15/DOX cells via LC-MS/MS. **(A)** PCA plot of HCT-15/DOX cells and HCT-15 cells in positive mode (left) and negative mode (right). **(B)** Volcano plot of differentially expressed metabolites in positive mode (left) and negative mode (right). The green and red dots indicate the significantly different downregulated and upregulated metabolites in HCT-15/DOX cells, respectively. **(C)** Metabolic pathway analysis of the differentially expressed metabolites in positive mode (left) and negative mode (right).

Metabolomic analysis of HCT-15 and HCT-15/DOX cells was subsequently carried out via LC-MS/MS. As shown in positive and negative ion modes, we identified 1533 and 1313 metabolites with known structural features, respectively. When we conducted principal component analysis (PCA), we detected obvious differences between the HCT-15/DOX and HCT-15 cells in both ion modes (**Figure 4A**). Our differential expression analysis revealed 654 differentially expressed metabolites, of which 555 were upregulated, 99 were downregulated in positive mode, and 445 metabolites, among which 357 were upregulated and 88 were downregulated in negative mode (**Figure 4B**). These results were based on the criteria of a fold change ≥ 1.5 or ≤ 0.67 and *p* < 0.05. Further details of the differentially expressed metabolites are presented in **Supplementary Tables S6** and **S7**. The results of the metabolic analysis revealed that metabolites involved primarily in the metabolism of pyrimidine, purine, amino sugar, and nucleotide sugars; ascorbate and aldarate; glycerophospholipid; vitamin B6; fructose and mannose; cysteine and methionine; and beta-alanine were differentially expressed (**Figure 4C**).

### 3.5 Combined proteomics and metabolomics analysis revealed altered mitochondrial metabolic characteristics associated with chemoresistance in colorectal cancer

**Figure 5:**
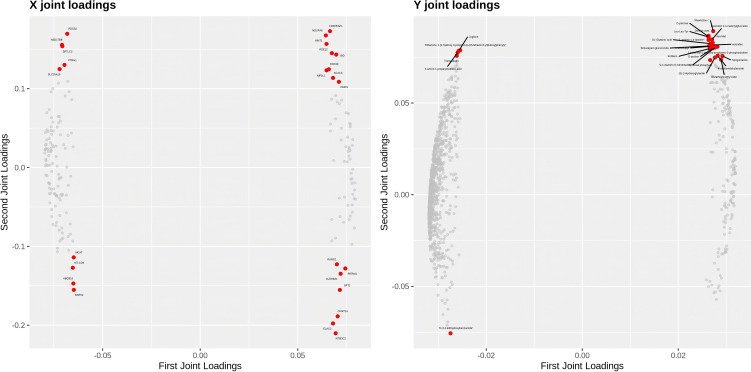
Integrated analysis plot of differentially expressed proteins and metabolites. An integrated analysis map of the proteomics and metabolomics of HCT-15/DOX cells and HCT-15 cells was created via the O2PLS (two-way orthogonal partial least squares) model. In the plot, proteins that are further from the origin are more closely related to metabolites, and vice versa. Highly correlated differentially expressed metabolites and proteins are shown as red nodes.

We used O2PLS (two-way orthogonal partial least squares) and joint pathway analysis methods to investigate the correlation between proteomics and metabolomics. The O2PLS analysis effectively delineated the correlation between proteomics and metabolomics, yielding high correlation coefficients (R2Xcorr = 0.981, R2Ycorr = 0.977). By utilizing the loading values of O2PLS, the joint analysis loading plot showing differentially expressed metabolites and proteins (as shown in **Figure 5**) revealed a robust correlation between them. **Figure 5** highlights the significant correlations of metabolites such as wuweizisu c, domoic acid, D-psicose, Leu-Leu-Tyr, quercetin 3-o-malonylglucoside, L-gulono-1,4-lactone, Asn-Met, DL-glutamic acid, and aclonifen with the differentially expressed proteins. Additionally, proteins such as NT5DC2, ELAC2, CKMT1A, CDK5RAP1, PDSS2, NDUFA6, GPT2, HSD17B8, and HINT1 were found to be closely related to differentially expressed metabolites.

**Figure 6:**
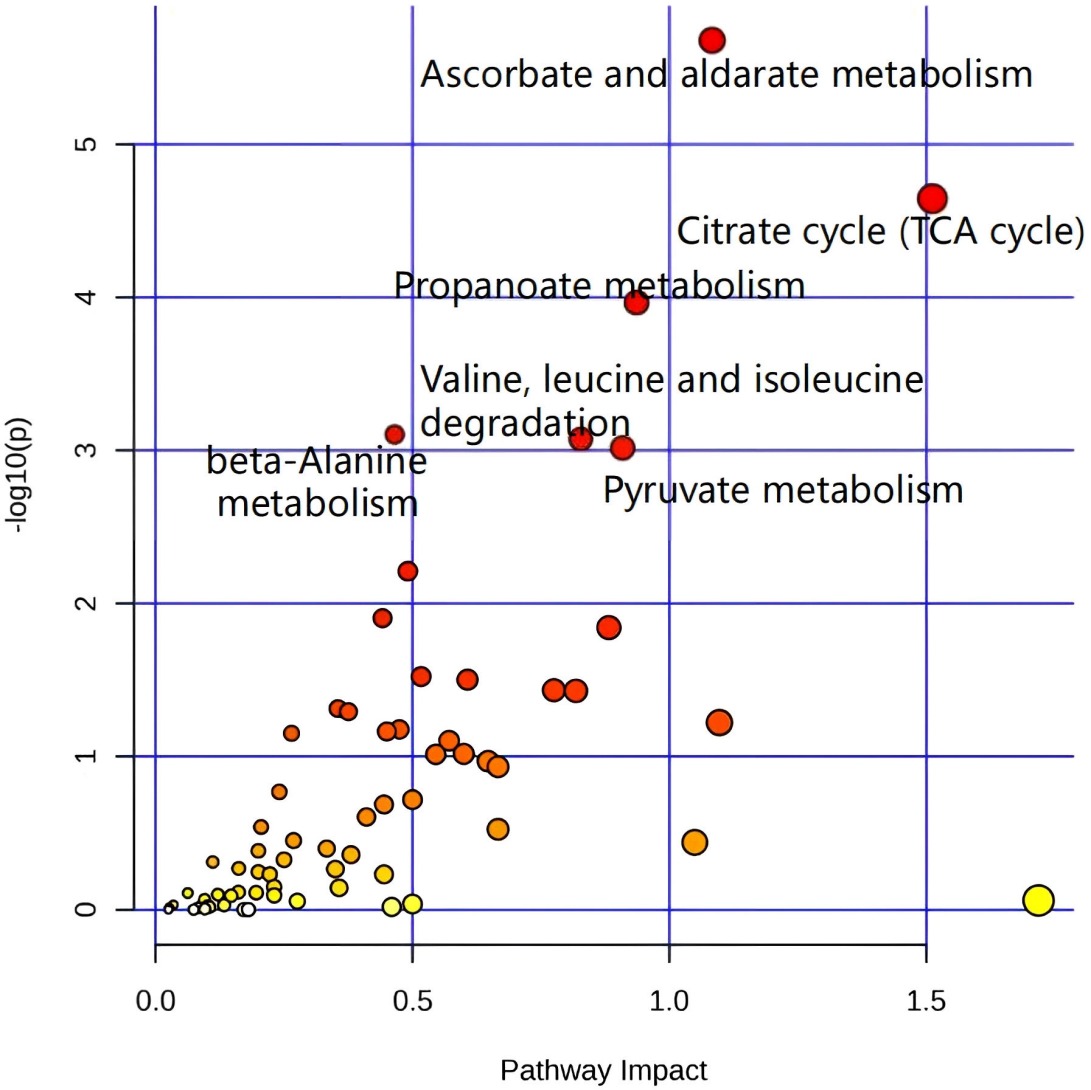
Integrated omics pathway analysis revealed that differentially expressed proteins and metabolites are involved in common biological processes.

Integrated omics pathway analysis suggested that differentially expressed proteins and metabolites are involved in common biological processes. Our results revealed that pathways associated with anabolic and catabolic metabolism (such as ascorbate and aldarate metabolism; propanoate metabolism; beta-alanine metabolism; valine, leucine and isoleucine degradation; and pyruvate metabolism); and energy synthesis (the TCA cycle) were affected primarily at both the proteome and metabolome levels (as shown in **Figure 6**). We also integrated our findings into a schematic model summarizing the core proteomic and metabolomic alterations underlying doxorubicin (DOX) resistance in colorectal cancer (CRC) cells (**Supplementary Figure S6**).

### 3.6 Treatment with the oxidative phosphorylation inhibitor oligomycin in HCT-15/DOX cells restored their sensitivity to chemotherapeutic agents

Pathway enrichment analysis of our multiomics data revealed that the tricarboxylic acid (TCA) cycle is a significantly dysregulated metabolic pathway. On the basis of these findings, we treated doxorubicin-resistant HCT-15/DOX cells along with the parental HCT-15 cells with oligomycin, an oxidative phosphorylation (OXPHOS) inhibitor. Notably, oligomycin treatment effectively restored doxorubicin sensitivity in resistant cells (**Supplementary Figures S7**, **S8A, B**; **Supplementary Table S1**).

Our proteomic analysis revealed the upregulation of succinate dehydrogenase complex subunit A (SDHA) in resistant cells. Since the CCK-8 assay primarily depends on cellular dehydrogenase activity for viability measurement, we employed the sulforhodamine B (SRB) assay—a dehydrogenase activity-independent method—to reassess cell proliferation and cytotoxicity. Using this alternative approach, we re-evaluated the differences in viability between doxorubicin-treated parental HCT-15 cells and resistant HCT-15/DOX cells (**Supplementary Figure S9**, **Supplementary Table S2**).

## 4 Discussion

Mitochondria are constantly changing organelles that meet the needs of the cell (20). These dynamic changes in their number and morphology can impact their function and contribute to disease. Changes in mitochondrial structure can involve alterations in crista morphology, mitochondrial DNA integrity, quantity, and dynamics, including fission and fusion (21, 22). Our research revealed that, compared with parental HCT-15 cells, HCT-15/DOX cells have a greater number of mitochondria, with increased maximum length and area, suggesting potential changes in mitochondrial function. Research indicates that in leukemia cells, an increase in mitochondrial fission results in decreased production of intracellular ROS and increased glycolysis, promoting chemoresistance in tumor cells (23). In breast cancer cells, mitochondrial fragmentation, along with an increasing number of mitochondria and higher levels of superoxide within mitochondria, leads to an increase in glycolysis, which contributes to tamoxifen resistance (24). Additionally, mitochondrial dynamics regulate the shape, size, number, energy metabolism, cell cycle, mitosis, and apoptosis of mitochondria (25). Mitochondrial metabolic dysfunction can lead to changes in dynamics, which in turn regulate mitochondrial integrity and function through feedback loops. This can worsen mitochondrial dysfunction and support the development and progression of tumors (26). Alterations in proteins related to mitochondrial dynamics can impact how sensitive tumor cells are to chemotherapy drugs (27). As a result, changes in the morphology and metabolic function of mitochondria in chemoresistant colorectal cancer cells are likely linked to chemotherapy resistance. Further proteomic and metabolomic analyses of both DOX-resistant HCT-15 cells and parental cells revealed different expression patterns in the resistant HCT-15 cells relative to the parental cells.

In a large-scale CRISPR knockout screening study, researchers systematically identified genetic drivers of chemotherapy resistance by targeting multiple cancer cell lines with seven chemotherapeutic agents. The results revealed that several chemotherapeutic drugs share common mechanisms of action, with the “cell cycle” function being closely related to oxaliplatin, irinotecan, and doxorubicin while having a lesser effect on other drugs. This suggests a certain degree of similarity in the mechanisms of action among these three chemotherapeutic agents (28). In another preclinical study, researchers employed an integrated metallomics/metabolomics approach for the first time to investigate oxaliplatin resistance in colon cancer. They identified distinct metabolic phenotypes between chemotherapy-sensitive and resistant cells, especially with respect to energy metabolism and fatty acid metabolism, which aligns with our findings (29). Furthermore, in a proteomic and transcriptomic study of patient-derived organoids from advanced colorectal cancer patients, researchers reported increased import of mitochondrial proteins engaged in ATP synthesis (such as ATP5A and ATP5B), along with elevated levels of citrate synthase, a key enzyme in the TCA cycle. These changes may indicate a metabolic shift from glycolysis to oxidative phosphorylation (OXPHOS) in tumors, potentially representing a mechanism of oxaliplatin resistance. Moreover, this energy supply process may effectively enable resistant tumor cells to repair oxaliplatin-induced DNA damage (30). Therefore, we hypothesize that there may be common mitochondrial-related metabolic pathways or targets underlying the resistance mechanisms to these chemotherapeutic drugs.

In our research on colorectal cancer chemoresistance, we identified certain differentially expressed mitochondrial proteins. Through analysis, we identified two important proteins: CPT1A and POLG. Our analysis suggested that lipid metabolism was a significantly affected pathway, with carnitine palmitoyltransferase I A (CPT1A) being highly expressed in HCT-15/DOX cells relative to that in HCT-15 parental cells. CPT1A is a vital enzyme in lipid metabolism and has a vital impact on fatty acid β-oxidation by facilitating the transfer of long-chain acyl groups from acyl-CoA to carnitine, which allows fatty acids to enter the mitochondrial matrix for oxidation (31). CPT1A is often overexpressed in different types of tumors and contributes to tumor metastasis, prevents apoptosis, and aids in resistance to radiotherapy and chemotherapy by enabling FAO (32–36). Moreover, research has demonstrated that reducing CPT1A levels in colorectal cancer cells can reverse their resistance to oxaliplatin by inhibiting FAO (37). High levels of CPT1A and increased FAO are crucial factors in tumor metastasis and drug resistance. Therefore, on the basis of the integration of our current findings with previous research evidence, we hypothesize that the upregulated expression of CPT1A in drug-resistant colorectal cancer cells may serve as a potential biomarker for chemotherapy resistance in this disease. Our study revealed that the expression level of mitochondrial DNA polymerase γ (POLG) was significantly elevated in drug-resistant colorectal cancer (CRC) cells and that high POLG expression in CRC was related to poor patient prognosis. Experimental validation demonstrated that the mtDNA copy number was markedly greater in drug-resistant cells than in their parental counterparts. POLG encodes DNA polymerase γ, the key enzyme responsible for mtDNA replication and repair in mitochondria, and its dysregulation has been closely associated with the development and progression of different cancers (38, 39). Previous studies have indicated that in pancreatic cancer, both the mRNA and protein expression levels of POLG are obviously greater in tumor tissues than in normal tissues and that high POLG expression is positively related to poor patient prognosis. Further mechanistic investigations revealed that the protumorigenic effect of POLG might be correlated with its aberrant regulation of mtDNA replication, thereby interfering with the mitophagy process (40). Moreover, multiple studies support the close association between mtDNA copy number variations and tumor chemosensitivity. For example, a reduction in mtDNA copy number can increase tumor cell sensitivity to cisplatin and doxorubicin (41). In breast cancer, doxorubicin-resistant cells presented a significant increase in mtDNA copy number, and targeted modulation of mtDNA copy number effectively reversed drug resistance (42). On the basis of these findings, we hypothesize that targeting the regulation of mtDNA copy number could become a promising strategy for reversing drug resistance in CRC, warranting further in-depth investigation.

In the present study, various mitochondrial-related metabolic changes were discovered between parental and resistant cells using untargeted metabolomics, proteomics, and integrated pathway enrichment analysis. These changes involved both anabolic and catabolic metabolism, such as pyrimidine metabolism, purine metabolism, ascorbate and aldarate metabolism, propanoate metabolism, and energy synthesis through the citrate cycle (TCA cycle). Research has indicated that disruption of these metabolic processes results in the progression of tumors. Tumor cells utilize pyrimidine metabolism to maintain a mesenchymal-like state driven through epithelial-mesenchymal transition (EMT), which enhances chemoresistance and stem-like characteristics (43). Changes in metabolic enzymes involved in purine metabolism may cause an imbalance in the purine pool, disrupting cell proliferation, migration, and death. Manipulation of purine metabolism can elicit antineoplastic effects through diverse mechanisms, including direct toxicity, modulation of the tumor microenvironment (TME), inhibition of DNA synthesis, and impairment of DNA damage repair (44). Perturbations in ascorbate and aldarate metabolism have been identified in renal cell carcinoma and ovarian cancer, possibly attributed to compromised energy metabolism in cancer cells. The administration of high-dose vitamin C, the bioactive form of ascorbate, has the potential to induce pro-oxidant effects and selectively eliminate cancer cells, thereby positioning this metabolic pathway as a potential therapeutic target to treat resistance in colorectal cancer (45–48). Dysregulation of propanoate metabolism can lead to the generation of pro-invasive characteristics in breast and lung cancer cells, increasing their metastatic potential (49). In a study by Hu C et al., methylmalonic acid (MMA) was identified in propanoate metabolites as a tumor metabolite in the progression of PanNEN on the basis of the serum metabolomics of patients with metastatic PanNEN and nonmetastatic PanNEN. A significant finding from this study was the potential role of MMA in triggering a new mechanism of EMT (50). The TCA cycle plays a central role in energy metabolism, macromolecule synthesis, and redox balance. The TCA cycle is a series of biochemical reactions that take place in the mitochondrial matrix. These reactions enable aerobic organisms to oxidize fuel sources and offer energy, macromolecules, and redox balance for cells (51). Certain key enzymes involved in the TCA cycle are associated with the occurrence and progression of cancer (52). Recent research suggests that cancer cells utilize the TCA cycle differently than normal cells do, which could increase the susceptibility of cancer cells to inhibitors that target reprogrammed metabolic pathways in the TCA cycle. We successfully restored doxorubicin sensitivity in chemoresistant colorectal cancer cells by applying the oxidative phosphorylation inhibitor oligomycin. As a result, targeting the TCA cycle with small molecule inhibitors that regulate the enzymes of the TCA cycle could be an effective approach for cancer treatment (53). Moreover, recent investigations have demonstrated that the development of tumor drug resistance involves mitochondrial dysfunction, characterized by a self-reinforcing cycle of L-lactate accumulation that significantly compromises oxidative phosphorylation efficiency (54). This work revealed that, compared with parental colorectal cancer cells, chemoresistant colorectal cancer cells exhibit changes in mitochondrial metabolic patterns. Targeting these metabolic processes may help overcome chemotherapy resistance in colorectal cancer.

## 5 Conclusion

In conclusion, this study highlights significant changes in the morphology and metabolic functions of mitochondria in colorectal cancer cells that are resistant to treatment compared with parental cells. Preliminary evidence indicates that CPT1A may be a potential biomarker for chemotherapy resistance in colorectal cancer and that targeted modulation of mtDNA copy number may represent an effective strategy to reverse such resistance. Multiomics integrated pathway enrichment analysis also revealed key alterations in metabolic processes such as pyrimidine metabolism, purine metabolism, ascorbate and aldarate metabolism, propanoate metabolism, and the citrate cycle (TCA cycle) in resistant cancer cells. These changes may affect the behavior of tumor cells and contribute to their resistance. Our research revealed that changes in mitochondrial metabolism are correlated with chemoresistance in colorectal cancer. We have also identified potential biomarkers for diagnosing or predicting colorectal cancer chemoresistance. Overall, this study improves our understanding of the ways in which mitochondrial metabolic changes contribute to chemoresistance in colorectal cancer and assists in the identification of potential targets for overcoming chemotherapy resistance in this type of cancer.

## Data Availability

The data analyzed in this study is subject to the following licenses/restrictions: The data presented in this study are available on request from the corresponding author. The data are not publicly available due to the further study required. Requests to access these datasets should be directed to zhengwei@xmu.edu.cn.
