# Correction: Multiomics analyses of human colorectal cancer reveal changes in mitochondrial metabolism associated with chemotherapy resistance

**DOI:** 10.3389/fonc.2025.1756306

**Published:** 2025-12-17

**Authors:** Shiyi Chen, Qian Li, Wei Zheng

**Affiliations:** 1 Department of Pathology, Zhongshan Hospital of Xiamen University, School of Medicine, Xiamen University, Xiamen, China; 2 School of Medicine, Xiamen University, Xiamen, China

**Keywords:** proteomics, metabolomics, colorectal cancer, chemotherapy resistance, mitochondrial metabolism

Affiliation “Department of Pathology, Zhongshan Hospital of Xiamen University, School of Medicine, Xiamen University, Xiamen, China” was omitted for author Shiyi Chen. This affiliation has now been added for author Shiyi Chen. Additionally, the order of affiliations needs to be adjusted. Affiliation “Department of Pathology, Zhongshan Hospital of Xiamen University, School of Medicine, Xiamen University, Xiamen, China” should be listed as the primary affiliation for the first author.

The correct Author affiliation is

“Shiyi Chen^1,2†^,Qian Li ^1†^ and Wei Zheng^1*^

^1^Department of Pathology, Zhongshan Hospital of Xiamen University, School of Medicine, Xiamen University, Xiamen, China, ^2^School of Medicine, Xiamen University, Xiamen, China”

The original version of this article has been updated.

