# Supplementary material for: Multiomics analyses of human colorectal cancer reveal changes in mitochondrial metabolism associated with chemotherapy resistance
Source: Front Oncol. 2025 Nov 10;15:1625797. doi: 10.3389/fonc.2025.1625797 (PMC12640853; doi:10.3389/fonc.2025.1625797)
Supplement: Supplementary file 1 [file DataSheet1.docx]

**Supplementary material**

Figure S1. The transmission electron microscopy images revealed an increased number of mitochondria in HCT-15/DOX cells compared with parental HCT-15 cells.


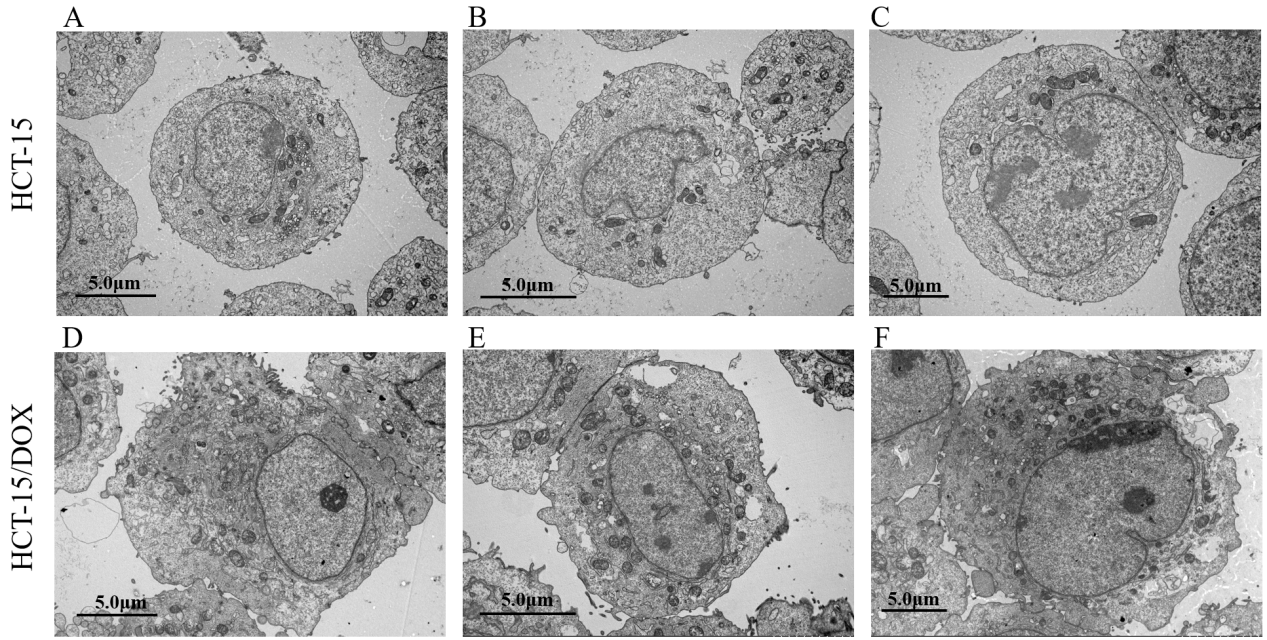


Figure S2. Electron microscopic analysis of mitochondrial morphology in HCT-15 and HCT-15/DOX cells. To quantitatively assess mitochondrial characteristics, we randomly selected five cells each from the HCT-15 and HCT-15/DOX cell lines for mitochondrial counting. Additionally, we measured the maximum length and cross-sectional area of ten mitochondria per cell line for statistical analysis.


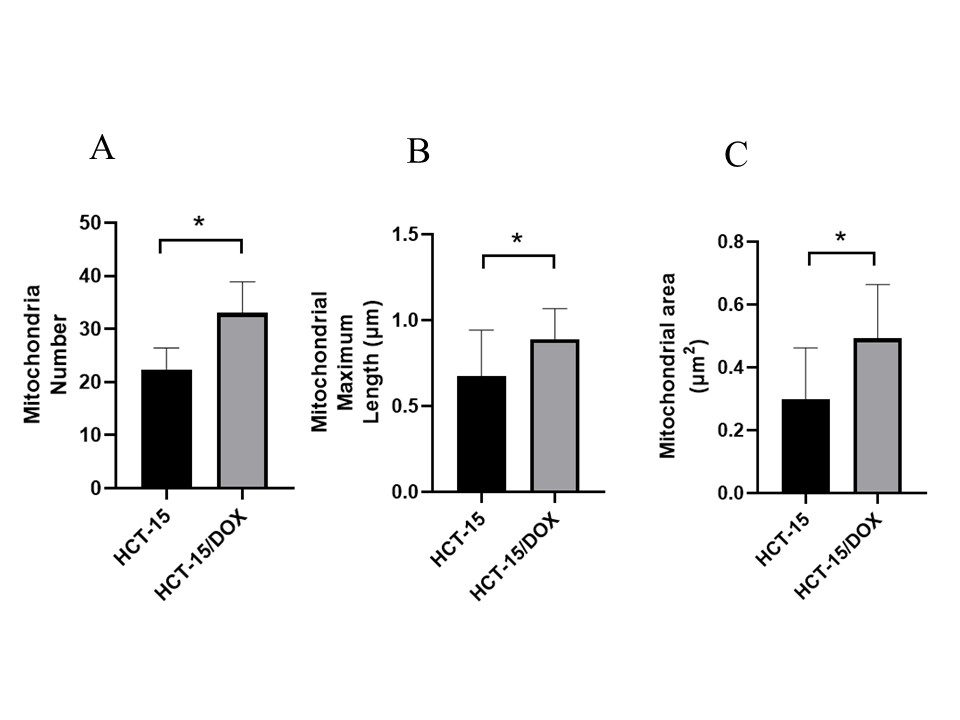


*P<0.05.

Figure S3.In proteomics, differentially expressed proteins (with a fold change >1.5 or <0.67 and a P-value <0.05) were subjected to KEGG pathway enrichment analysis.


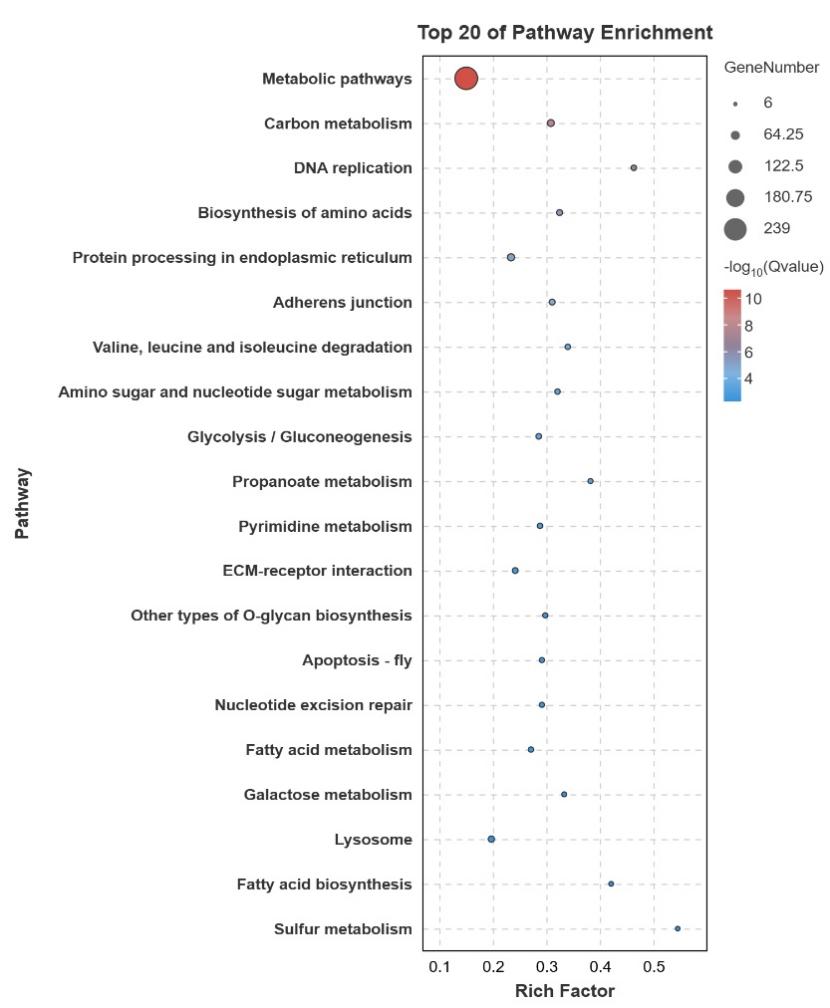


Figure S4. Kaplan-Meier curves of overall survival time for CPT1A （CPT1A, also known as CPT1, CPT1-L, or L-CPT1.）and POLG.


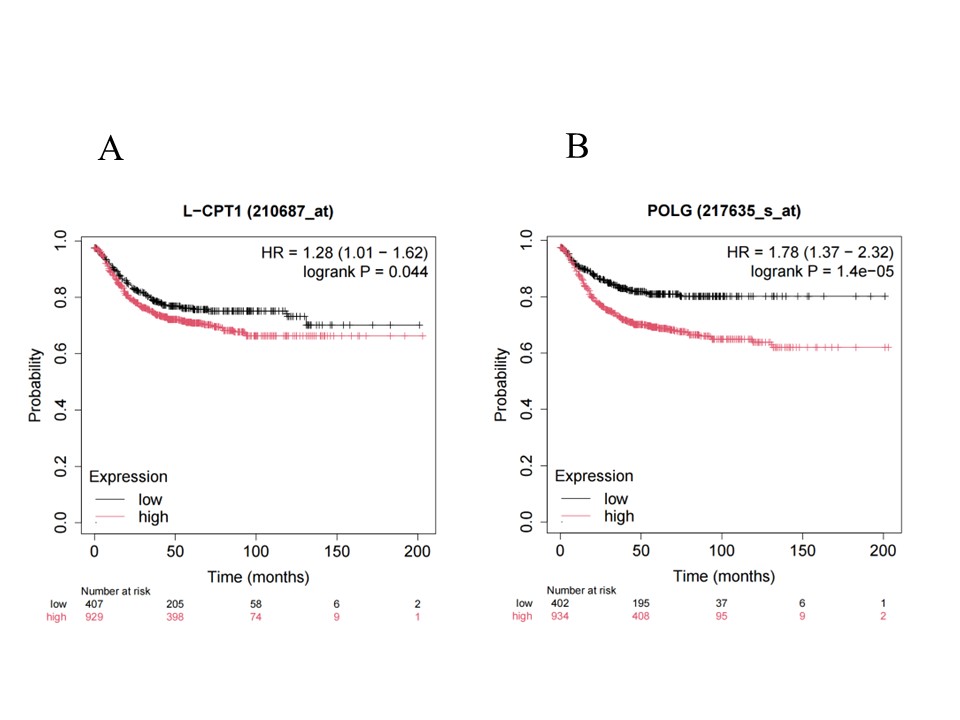


Figure S5. Mitochondrial DNA copy number in HCT-15 and HCT-15/DOX cells.


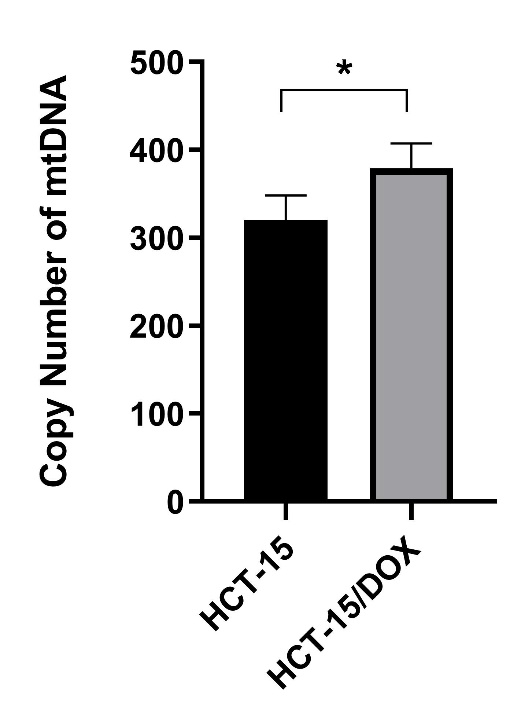


The mitochondrial DNA copy number was represented by the average of 2ΔCt values, calculated as the difference in cycle thresholds between ND1/SLCO2B1 and ND5/SERPINA1. *p-value < 0.05. Data are presented as mean ± SD; n = 4. Statistical significance was determined using Student's t-test.

Figure S6. The schematic that summarizes the core proteomic and metabolomic alterations underlying DOX resistance in CRC cells.


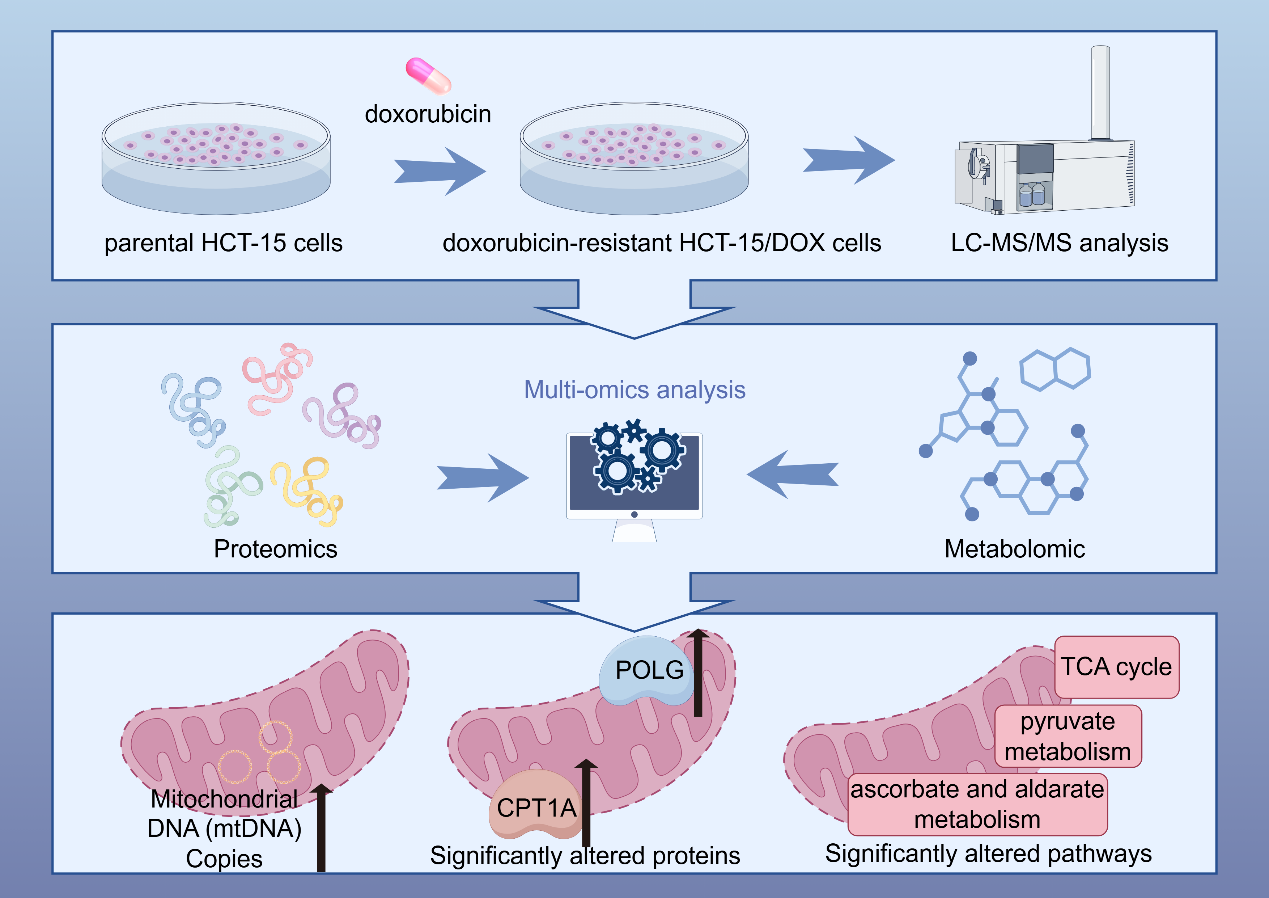


Figure S7. Oligomycin enhance the sensitivity of HCT-15/DOX cells to Doxorubicin. Both HCT-15/DOX cells treated with DMSO and HCT-15/DOX cells treated with Oligomycin were exposed to Doxorubicin at concentrations of 0, 0.25, 1, 4, 16, and 64 µg/mL for 48 hours. Afterward, the Sulforhodamine B (SRB) Assay was performed. Points indicate mean values; lines stand for standard deviation (n = 5).


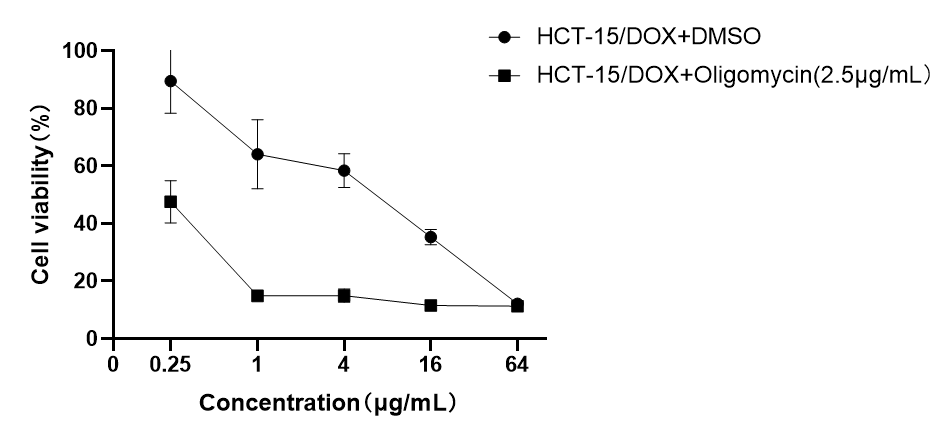


Figure S8. Oligomycin enhance the sensitivity of HCT-15 cells to Doxorubicin. Both HCT-15 cells treated with DMSO and HCT-15 cells treated with Oligomycin were exposed to Doxorubicin at concentrations of 0, 0.25, 1, 4, 16, and 64 µg/mL for 48 hours. Afterward, the Sulforhodamine B (SRB) Assay was performed. Points indicate mean values; lines stand for standard deviation (n = 5).


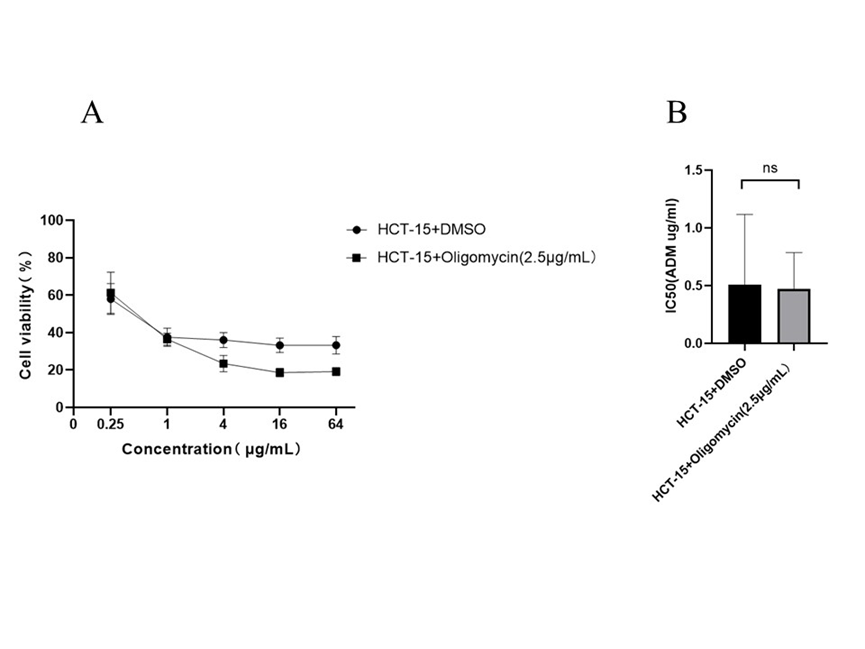


Figure S9. The viability of HCT-15 and HCT-15/DOX cells subject to treatment with Doxorubicin was assessed through the Sulforhodamine B (SRB) Assay. The HCT-15/DOX cells were less sensitive to Doxorubicin than the parental cells. Both HCT-15 and HCT-15/DOX cells were exposed to Doxorubicin at concentrations of 0, 0.25, 1, 4, 16, and 64 µg/mL for 48 hours. Afterward, the Sulforhodamine B (SRB) Assay was performed. Points indicate mean values; lines stand for standard deviation (n = 5).


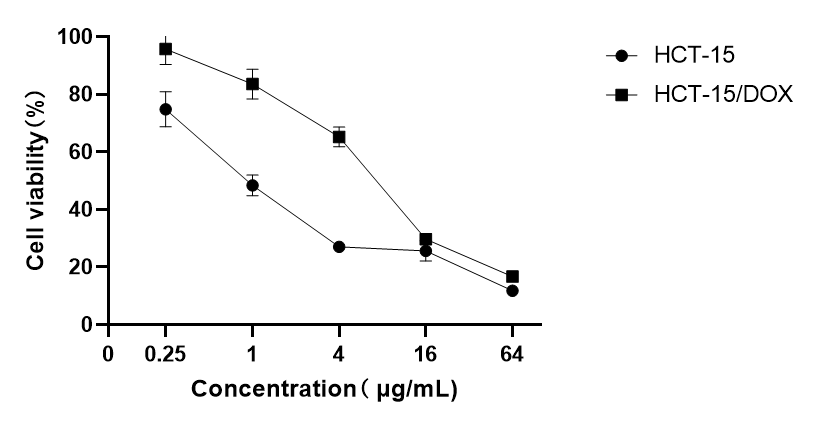


**Materials and methods**

**Compounds**

Oligomycin（TargetMol, USA), an antifungal antibiotic, is an inhibitor of H+-ATP synthase. Oligomycin can block oxidative phosphorylation and the electron transport chain.The compounds were dissolved in dimethyl sulfoxide (DMSO; MP Biomedicals, Solon, OH, USA), and the concentration of DMSO in the cell cultures was ensured to be less than 0.1% during the experiments.

**Sulforhodamine B (SRB) Assay**

Collect logarithmic-phase cells, adjust the cell suspension concentration, and aliquot into a 96-well plate at 100 μL per well. Incubate the plate at 37°C with 5% CO₂ to allow cell attachment, and culture for 6–24 hours. After incubation, remove the plate, discard the culture medium, and wash the cells 1–2 times with PBS. Add 100 μL of pre-cooled fixative (Yeasen, Shanghai, China) to each well, and let it stand at room temperature for 5 minutes, followed by incubation at 4°C for 1 hour. Discard the supernatant, wash three times with Wash Buffer 1 (Yeasen, Shanghai, China), and air-dry at room temperature. Add 100 μL of staining solution (Yeasen, Shanghai, China) to each well, and incubate in the dark for 20 minutes (the plate can be wrapped in aluminum foil and gently shaken on a horizontal or rocking shaker). After incubation, discard the staining solution and wash five times with Wash Buffer 2 (Yeasen, Shanghai, China) (perform quickly to prevent leakage of the staining solution from the cells), ensuring that residual staining solution is thoroughly removed, and air-dry at room temperature. Add 200 μL of solubilization buffer (Yeasen, Shanghai, China) to each well, and incubate in the dark for 30 minutes (the plate can be wrapped in aluminum foil and gently shaken on a horizontal or rock-ing shaker). Finally, measure the absorbance at a wavelength of 515 nm.

**Mitochondrial DNA (mtDNA) Copies**

The number of mtDNA copies was determined by targeting four distinct genes: NADH dehydrogenase subunit 1 (ND1), solute carrier organic anion transporter family member 2B1 (SLCO2B1), NADH dehydrogenase subunit 5 (ND5), and serpin family A member 1 (SERPINA1), using the Human mtDNA Monitoring Primer Set (Cat. #7246, Takara Bio, Tokyo, Japan). Genomic DNA was extracted from cell samples using a DNA extraction kit (Cat. #RK30110, ABclonal, Wuhan, China). For the subsequent reaction, a mixture was prepared containing the extracted genomic DNA, four specific primers from the Human mtDNA Monitoring Primer Set, and a PCR enzyme (SYBR Premix Ex Taq™ II, Cat. #RR820A, Takara Bio, Tokyo, Japan). The quantitative PCR amplification was performed using a Roche LightCycler® 480 system under the following thermal cycling conditions: an initial denaturation step at 95 °C for 30 s, followed by 40 cycles of denaturation at 95 °C for 5 s and annealing/extension at 60 °C for 30 s. The mtDNA copy number was calculated as the mean of 2ΔCt values derived from the cycle threshold (Ct) differences between ND1/SLCO2B1 and ND5/SERPINA1.

**Analysis Based on Public Databases**

Survival analysis was performed using KM-plotter（https://kmplot.com/analysis/），differential gene expression analysis between tumor and normal tissues was performed using TNMplot（https://tnmplot.com/analysis/）.
